# Propagation and domains of the invariant ion-acoustic solitons in the plasmas

**DOI:** 10.1038/s41598-024-54263-x

**Published:** 2024-02-13

**Authors:** E. Saberian

**Affiliations:** Department of Physics, Faculty of Basic Sciences, University of Neyshabur, 9319774446 Neyshabur, Iran

**Keywords:** Solitary waves in space plasmas, Invariant kappa distribution, Polytropic index, Space physics, Plasma physics, Statistical physics, thermodynamics and nonlinear dynamics, Space physics, Plasma physics, Statistical physics, thermodynamics and nonlinear dynamics

## Abstract

Observational pieces of evidence of space probes Voyagers and IBEX to study the Sun’s heliosphere, the outer Solar system, and interstellar space beyond the Sun’s heliosphere indicate that perturbations in some regions may occur in situations out of the pure thermal equilibrium, e.g., in the outer heliosphere regions, the inner heliosheath regions, and in heliopause regions. The data analysis extracted from these probes also shows that the transitions between the near/far-equilibrium states may happen in some areas, e.g., the slow solar wind $\mathrm {e^{-}}$ (Ulysses) plasmas and the fast solar wind $\mathrm {He^{+}}$ plasmas. The modern formalism of the kappa distributions explains the distinction between the near/far-equilibrium states under the value of the kappa index, as an intensive thermodynamic parameter. For providing more clarity to this formalism, an invariant kappa index as the zero dimensionality spectral index $\kappa _{0}$ is determined to consider the physical and thermodynamic feature of the kappa index in space plasmas, where it is independent of the dimensionality, the degrees of freedom, or the numbers of particles. Recently, this idea has extended for studying the invariant ion-acoustic waves (IAWs) in the astrophysical plasmas. Then, we discussed the pure thermodynamic features of the background particles. By utilizing $\kappa _{0}$, we found the distinction of the involved IAWs diagrams in the near/far-equilibrium states and also the transition from far-equilibrium states to the near-equilibrium states in the vicinity of a critical spectral/polytropic index. This paper extends the invariant formalism of the ion waves to the propagation features and structure of the nonlinear perturbations in the outer Solar system and interstellar space beyond the Sun’s heliosphere relevant to the mentioned observational evidences. We study the propagation and allowed domains of the invariant ion-acoustic solitary waves (IASWs) by considering the advanced aspects of the kappa distribution formalism. The central parameters of our formalism for analysis of the allowed domains of the solitary waves and shocks are the polytropic (adiabatic) index associated with the kappa distributed electrons, $\gamma _{e}$, and a well-defined and extended Mach number ${\mathcal {M}}_{\gamma _{e}}$ (the fractional wave speed to the generalized ion-sound speed). We have used Sagdeev’s methodology for deriving the energy-integral equation of the IASWs, which describes the formation of the possible potential wells (pseudo-potentials) for trapping the arbitrary amplitude solitons (pseudo-particles). The analysis of the Mach number domains is developed by extracting $(\phi ,{\mathcal {M}}_{\gamma _{e}})$ domains for the possibility of the solitary wave solutions in the plasma. We also show variation of the relevant $(\gamma _{e},{\mathcal {M}}_{\gamma _{e}})$ domains. The formalism of the energy-integral equation and the domains of invariant IASWs has illustrated in two cases. At first, we show the general aspects of the problem by considering $T_{i} \ll T_{e}$ (the cold ion plasma limit), and then we extend it to a warm plasma with finite-temperature ions.

## Introduction

The observational data of spacecraft confirm the particle’s velocity distribution in the space plasmas have the non-Maxwellian tails^1–4^, where they decrease as a power law distribution with the particle speed, known as the supra-thermal tails. A well-known model that could describe these particles is the kappa ($\kappa$) distribution formalism, which introduced by Vasyliunas in 1968^5^ for describing particles in plasmas out of the thermal equilibrium such as the Magnetosphere environment and Solar winds. The proposed distribution of Vasyliunas was a power-law generalization of the Maxwell-Boltzmann distribution function. At present, we know that the systems with long-range interactions and correlation, such as the plasmas, may be appropriately described by the *q* non-extensive Tsallis formalism^6,7^, which proposed by Tsallis in 1988 as a generalization of the Boltzmann-Gibbs statistics^8^. There is a close connection between the $\kappa$-distribution function and the *q*-distribution function of the Tsallis statistics, e.g., Livadiotis and McComas have shown how the kappa distributions arise naturally from the Tsallis statistical mechanic^9^. Detailed information on the historical background and formalism of the kappa distribution, its connection with the Tsallis statistics, and its applications in the space plasmas have reviewed by Livadiotis^10^.

Using the $\kappa$- or *q*-distribution functions, the plasma waves, oscillations, instabilities, and the other aspects of space plasmas may be studied in the extended formalisms. The spectral indices $\kappa$ and *q* is considered a measure of quantifying the stationary states of the space plasma. Pierrard and Lazar have shown that $\kappa$ index may describe the thermodynamic distance of the system from the thermal equilibrium^11^. For example, the influence of pick-up ions on space plasma distribution shows that the addition of highly ordered distributions of pick-up ions can increase the ordering of space plasmas, decreasing their entropy, and driving them away from equilibrium^12^.

We note that the modern formalism of the kappa distribution connects to the zeroth law of thermodynamics and the thermal equilibrium, so it is allowed to be parameterized by temperature^13^. The kappa distributions correspond to the generalized thermal equilibrium, where correlations may exist. In contrast, in the classical thermal equilibrium, no correlations exist among the particles^13^. Furthermore, it has recently proved that the thermodynamics of particles’ physical correlations are consistent only with the existence of kappa distribution^14^.

The linear/nonlinear aspects of the wave propagation in space and astrophysical plasmas have been widely studied in the context of the Tsallis non-extensive statistics and/or the kappa distribution formalism, e.g., the plasma oscillations in a collision-less electron-ion plasma^15,16^, the ion-acoustic waves (IAWs) in a collision-less magnetic-field-free plasma^17^, the ion plasma waves in a pure pair-ions plasma or equivalently the plasma oscillations in a collisionless electron-positron plasma^18–20^, the arbitrary amplitude ion-acoustic solitary waves (IASWs) in a two-component plasma^21^, the ion-acoustic double layers in a two-component plasma^22^, the IASWs in an electron beam-superthermal plasma^23^, the nonlinear dust-acoustic solitons in multi-component space plasmas^24,25^, the ion-acoustic solitons in solar winds plasma with superthermal electrons^26^, the generalized formalism of the plasma sheaths in a kappa-distributed plasma^27^, and the maximal Mach number for solitons in a collision-less warm electron-ion plasma, where the isothermal and adiabatic models of the ion-sound waves have been considered^28^.

Note that there are other proposed models for describing the non-Maxwellian features of the plasmas in space, such as the non-thermal alpha ($\alpha$) model advanced by Cairns et al.^29^ which introduced at first for explanation of the solitary electrostatic structures involving density depletions that have been observed in the upper ionosphere in the auroral zone by the Freja satellite^30^. This model has some applications for studying the particle trapping in the plasma (see e.g. Ref.^31^).

An exciting feature of the Tsallis non-extensive statistics is that the spectral indices (*q* and $\kappa$) of the canonical distribution function depend on the numbers of degrees of freedom or dimensionality^32,33^, where *q* or $\kappa$ is related to the correlation between the system’s particles. We have studied the dimensional dependency of the plasma oscillations on the number of degrees of freedom (involved in the spectral indices of the non-extensive statistical mechanics) by using the escort (modern) formalism of the canonical probability distribution^34^. Note that the ordinary (old) formalism of the canonical probability distribution has some physical inconsistencies that have solved by introducing the escort probability distribution and some other constraints^9^. In summary, the advantages of the escort formalism are as follows: it is independent of an energy level; it provides the correct and consistent partition of the system’s internal energy to the subsystem’s partial internal energies, and it is compatible with a meaningful temperature^35^.

The formalism of the distribution function of the plasma and the involved spectral index therein is dependent on the number of degrees of freedom *d*, from the equilibrium state, where $\kappa _{d}\rightarrow \infty$ and $q_{d}\rightarrow 1$, to the anti-equilibrium state, where $\kappa _{d}\rightarrow \frac{d}{2}$ and $q_{d}\rightarrow 1+\frac{2}{d}$, and in all the intermediate states^32^. Here, $\kappa _{d}$ and $q_{d}$ are *d*-dimensional spectral indices. The notion of the invariant spectral index may resolve some inconsistencies that may arising from applying the *d*-dimensional canonical probability distribution function. By defining the invariant spectral indices as the zero dimensionality spectral indices, $\kappa _{0}$ or $q_{0}$, which are independent of the dimensionality, the degrees of freedom, or the number of particles, it is possible to consider separately the physical features of the spectral index^32^. We mention that the *d*-dimensional index $\kappa _{d}$ depends on the invariant index $\kappa _{0}$ by the relation $\kappa _{d}=\kappa _{0}+\frac{d}{2}$^32^. For the interested reader, the general formalism of the escort canonical probability distribution in terms of the invariant spectral index $\kappa _{0}$ and the resultant number density for the kappa distributed particles exist in the Supplementary Material file.

Determining the Mach number domain is one of the challenges in studying the nonlinear structures in the plasmas, such as the solitons, shock waves, and double layers. The Mach number ${\mathcal {M}}$ is defined as the fractional wave speed to the ion-sound speed in the plasma. We note that the definition of the Mach number and its domains in the plasma sheaths is another problem (The interested reader may refer to Ref.^27^). The flawed normalization of the soliton speed (and also the other normalized parameters) may lead to inaccurate solutions for the Mach number domains, as Dubinov has described it as “a widespread inaccuracy in defining the Mach number of solitons in a plasma”^36^. To find the accurate solution of the Mach number domains in the propagation of the IASWs, we have to consider the complete formulations of the Debye screening length and the ion-sound speed in defining the normalized parameters. In the modern kappa distribution formalism, the generalized formulations of the Debye length^37^ and the ion-sound speed^38^ depend strongly on the stationary state of the plasma by the functional dependency on the extended polytropic index $\gamma$. Generally, the formulations of the ion-sound speed and Debye length are not unique. Still, they also depend on other parameters of the plasma, such as the density and the temperature of the constituents of the plasma, and they depend on the stationary state of the plasma.

By considering all these issues, the general aspects of the invariant ion-acoustic waves in the space plasma have been recently studied^39^, by using the kinetic Vlasov-Poisson equations in the linear regime and the hydrodynamic fluid equations both in the linear and nonlinear regimes. We discussed the solitary wave solutions of the invariant ion-acoustic waves using the perturbation technique, which is suitable for the small amplitude IASWs at speeds around 1 Mach. In the present study, we want to study the propagation and the allowed domains of the arbitrary amplitude invariant IASWs, which implies applying Sagdeev’s pseudo-potential method^40^. In this technique, we may derive an energy-integral equation that describes the trapping of the solitons (the pseudo-particles) in the typical potential wells (the pseudo-potentials). We also analyze the Mach number domains using the pseudo-potential function in detail.

Recently, we introduced a different viewpoint of Sagdeev’s methodology based on the analysis of the Mach number domains corresponding to the involved potential, whereby considering the overlap of all the constraints for the formation of the pseudo-potential, the allowed $(\phi ,{\mathcal {M}})$ domains of the arbitrary amplitude IASWs and the possible double layers (DLs) are presented^41^. On the other hand, in the commonly used analysis of the allowed Mach domains, the infinite compression limit, labeled with a critical potential $\phi _{cr}$, is considered the maximum width of the possible potential wells. Then, one may derive the upper limit of the Mach number by using this critical potential, besides the threshold Mach number limit for possible excitation of the IASWs^40^. In this study, we will apply both methods for studying the domains of the arbitrary amplitude invariant IASWs, where both of them have their advantages.

The structure of this paper is as follows: First, we introduce the model equations and also the normalization of the parameters in terms of the generalized formalisms of the ion-sound speed and Debye length. Then, we review the methods for the linear and nonlinear analysis of the IASWs, where the general aspects of the Sagdeev’s methodology will be presented together with the criteria for trapping the IASWs and possible DLs in the plasma; By using the linear analysis, we show the dispersion relation of the IASWs, where it confirms the generalized ion-sound speed in space plasmas; Then, we examine the fully nonlinear analysis of the invariant IAWs, where we will derive the solitary wave solutions in two cases, i.e., the cold-ion plasma and the warm-ions plasma with a finite temperature; We also show a detailed analysis of the domains of the extended (adiabatic) Mach number; Finally, we summarize the conclusions of this study.

## The model equations

For deriving the energy-integral equation of the invariant IASWs in the plasma, we need the set of hydrodynamic equations for the ions in one dimension (corresponding to the direction in which the compression/rarefaction of the ion oscillations would propagate) as follows

1a $$\begin{aligned} \frac{\partial n_{i}}{\partial t} + \frac{\partial (n_{i}v_{i}) }{\partial x}=0, \end{aligned}$$

1b $$\begin{aligned} \left( \frac{\partial v_{i}}{\partial t} + v_{i} \frac{\partial v_{i}}{\partial x} \right) = -\frac{ Z_{i}e}{ m_{i}} \frac{\partial \phi }{\partial x} - \frac{1}{ m_{i} n_{i}} \frac{\partial p_{i}}{\partial x}, \end{aligned}$$

1c $$\begin{aligned} \frac{p_{i}}{n_{i}^{\gamma _{i}}}=const. , \end{aligned}$$

1d $$\begin{aligned} \varepsilon _{0} \frac{\partial ^{2} \phi }{\partial x^{2}}=-e(Z_{i}n_{i}-n_{e}), \end{aligned}$$

where $n_{i}$, $v_{i}$ and $p_{i}$ are the number density, fluid velocity, and the pressure of the ions, respectively, $\gamma _{i}$ is the polytropic (adiabatic) index in thermodynamic evolution of the ions, $\phi$ is the electrostatic potential of the ion waves, and $n_{e}$ is the number density of electrons. Here, $Z_{i}$ denotes the number of charges of the ions that depends on the atomic number of the ions. For example, $Z_{i}=1$ represents a Hydrogen plasma ($H^{+1}$ ions) and $Z_{i}=2$ denotes a Helium plasma ($He^{+2}$ ions).

The electrons are imposed on the electrostatic potential of the ion waves when they are pulled by the compression/rarefaction of the ions. So, the potential energy of the electrons in the electrostatic potential of the ions is $\Phi _{e}=-e\phi (x)$ and the number density of the kappa distributed electrons (see the Supplementary Material) is written as

2 $$\begin{aligned} n_{e}(x)=n_{\infty ,e} \cdot \left[ 1-\frac{1-\gamma _{e}}{\gamma _{e}}\cdot \frac{e\phi (x)}{k_{B}T_{\infty ,e}}\right] ^{\frac{1}{\gamma _{e}-1}}, \end{aligned}$$

where $n_{\infty ,e}$ and $T_{\infty ,e}$ are the number density and the temperature of the electrons at infinity (where the potential is zero), and $\gamma _{e}$ is the polytropic index associated with the kappa distributed electrons as $\gamma _{e}=\frac{\kappa _{0}+\frac{1}{2}d_{\Phi ,e}}{\kappa _{0}+1+\frac{1}{2}d_{\Phi ,e}}$. Here, $d_{\Phi ,e}$ is the potential degrees of freedom for the electrons in the presence of the ion waves’ potential and it is given by the formula $\frac{1}{2}d_{\Phi ,e}= -\frac{e\langle \phi (x)\rangle }{k_{B}T_{\infty ,e}}$. Note that if $d_{\Phi ,e}$ is positive, then $\gamma _{e}$ is less than one, and if it is negative, then $\gamma _{e}$ can be either larger or smaller than one^42^. Noting that the ion waves’ potential (as opposed to the potential at infinity) is positive, $\phi >0$, so $d_{\Phi ,e}$ is negative and then $\gamma _{e}$ may be either larger or smaller than one.

In this formalism, we have two sub-regions, i.e., the far-equilibrium regions, in which $0<\gamma _{e}<0.5$; and the near-equilibrium areas in which $0.5<\gamma _{e}<1$. Here, the stationary state with the polytropic index $\gamma _{e}=0.5$ denotes the escape state of the system, where the system can escape from the far-equilibrium regions toward the near-equilibrium regions^32,39^. Two asymptotic limits in this notation are the equilibrium state ($\gamma _{e}\rightarrow 1$) and the anti-equilibrium state ($\gamma _{e}\rightarrow 0$), where the distribution function collapses. The far-equilibrium regions indicate the distributions with high energy tails, where more supra-thermal particles exist in the plasma.

We use a set of well-defined normalized parameters as follows

3 $$\begin{aligned} \frac{x}{\lambda _{D,\gamma _{e}}}\rightarrow x^{'}, \; \; \; \frac{t}{\omega _{pi}^{-1}}\rightarrow t^{'}, \; \; \; \frac{v_{i}}{c_{s,\gamma _{e}}}\rightarrow v^{'}, \; \; \; \frac{n_{i}}{n_{\infty ,i}}\rightarrow n^{'}, \; \; \; \frac{p_{i}}{n_{\infty ,i}k_{B}T_{\infty ,i}}\rightarrow p^{'}, \; \; \; \frac{e\phi }{k_{B}T_{\infty ,e}}\rightarrow \phi ^{'}, \; \; \; \end{aligned}$$

where, $\lambda _{D,\gamma _{e}}=\sqrt{\gamma _{e}\frac{\varepsilon _{0}k_{B}T_{\infty ,e}}{e^{2}n_{\infty ,e}}}$ is the generalized Debye length via the kappa distributed electrons^37^, $\omega _{pi}=\sqrt{\frac{Z_{i}^{2}e^{2}n_{\infty ,i}}{\varepsilon _{0}m_{i}}}$ is the ion oscillation frequency, and $c_{s,\gamma _{e}}=\sqrt{\gamma _{e}\frac{Z_{i}k_{B}T_{\infty ,e}}{m_{i}}}$ is the generalized ion-sound speed of the plasma by the kappa distributed electrons^38^. Furthermore, $n_{\infty ,i}$ is the number density of the ions at infinity, where satisfies the quasi-neutrality conditions of the plasma as $Z_{i}n_{\infty ,i}=n_{\infty ,e}$. A simple relation exists between the ion oscillation frequency, the generalized Debye length, and the generalized ion-sound speed as $\omega _{pi}\cdot \lambda _{D,\gamma _{e}}=c_{s,\gamma _{e}}$^38^. In the asymptotic limit, $\gamma _{e}\rightarrow 1$, the classical relation $\omega _{pi}\cdot \lambda _{D,\infty }=c_{s,\infty }$ has been retained between the classical parameters, where the $\lambda _{D,\infty }=\sqrt{\frac{\varepsilon _{0}k_{B}T_{\infty ,e}}{e^{2}n_{\infty ,e}}}$ and $c_{s,\infty }=\sqrt{\frac{Z_{i}k_{B}T_{\infty ,e}}{m_{i}}}$ are the classical (Maxwellian) Debye length and the ion-sound speed, respectively. Then, the normalized equations for the propagation of the IASWs are written as

4a $$\begin{aligned} \frac{\partial n^{'}}{\partial t^{'}} + \frac{\partial (n^{'}v^{'}) }{\partial x^{'}}=0, \end{aligned}$$

4b $$\begin{aligned} \frac{\partial v^{'}}{\partial t^{'}} + v^{'} \frac{\partial v^{'}}{\partial x^{'}} = -\frac{1}{\gamma _{e}} \frac{\partial \phi ^{'}}{\partial x^{'}} - \frac{\sigma _{ie}}{ Z_{i}} \frac{\gamma _{i}}{\gamma _{e}} {n^{'}}^{\gamma _{i}-2} \frac{\partial n^{'}}{\partial x^{'}}, \end{aligned}$$

4c $$\begin{aligned} \frac{\partial ^{2}\phi ^{'}}{\partial {x^{'}}^{2}}= \gamma _{e} \left[ \left( 1-\frac{1-\gamma _{e}}{\gamma _{e}} \phi ^{'} \right) ^{\frac{1}{\gamma _{e}-1}}-n^{'} \right] , \end{aligned}$$

where $\sigma _{ie}=\frac{T_{\infty ,i}}{T_{\infty ,e}}$ is the fractional temperature of the ions to electrons at the infinity. We have combined the momentum transfer equation and the pressure evolution equation in Eq. (4b).

## Methods

### The linear analysis

By linearizing the Eqs. (4a)–(4c), assuming that the perturbed variables oscillate as $exp[i(\mathbf {k^{'}}\cdot \textbf{X}-\omega ^{'} t)]$, where $\mathbf {k^{'}}$ and $\omega ^{'}$ are the normalized wave vector and wave frequency, respectively, and $\textbf{X}$ is the position vector, then by simultaneously solving the linearized equations and neglecting the terms of the second and higher orders, we may find a linear dispersion relation as follows

5 $$\begin{aligned} \frac{{\omega ^{'}}^{2}}{{k^{'}}^{2}}=\frac{1}{1+{k^{'}}^{2}}+\frac{\gamma _{i}\sigma _{ie}}{Z_{i}\gamma _{e}}. \end{aligned}$$

Noting the normalization given in relations (3), the linear dispersion relation is written in terms of the original parameters as

6 $$\begin{aligned} \frac{{\omega }^{2}}{{k}^{2}}=\frac{1}{1+(k \lambda _{D,\gamma _{e}})^{2}}\frac{\gamma _{e}Z_{i}k_{B}T_{\infty ,e}}{m_{i}}+\frac{\gamma _{i}k_{B}T_{i}}{m_{i}}. \end{aligned}$$

where we have used the inverse transformation as $\omega ^{'} \rightarrow \frac{\omega }{\omega _{pi}}, \; k^{'} \rightarrow k \lambda _{D,\gamma _{e}}$. The adiabatic index of the ions may be considered as $\gamma _{i}=3$ for the compression/rarefaction of the ions in one dimension ($d_{i}=1$). This result agrees with the ones in the earlier studies^38,39^. It has proven that the invariant ion-sound speed increases for the higher adiabatic indices, where it tends to the maximum phase speed of IAWs at the isothermal limit $\gamma _{e}\rightarrow 1$^39^.

Note that Eq. (6) is the standard dispersion relation formalism of the IAWs in the plasma with observational shreds of evidence in laboratories. For example, the experimental examination of the electrostatic waves in a pure pair-ion plasma (containing the fullerenes $C_{60}^{-}$ and $C_{60}^{+}$), have been reported by Oohra et al.^43^, where properties of the wave propagation along the B-field lines have measured. In this study, three electrostatic modes in the estimated dispersion relation have been reported with frequencies as $\frac{\omega }{2\pi }<8kHz$ (lower frequency band), $8kHz<\frac{\omega }{2\pi }<32kHz$ (intermediate-frequency band), and $\frac{\omega }{2\pi }>32kHz$ (higher frequency band), where they are respectively corresponding to the ion-acoustic waves (IAWs), the backward intermediate-frequency waves (IFWs) which are the ion cyclotron waves, and the ion plasma waves (IPWs) or Langmuir waves. The formalism of dispersion relation described by Eq. (6) corresponds to the low-frequency band of IAWs.

### The nonlinear analysis

For nonlinear analysis of the invariant IAWs, we use Sagdeev’s pseudo-potential approach with some modified constraints as presented in detail in Ref.^41^. Here, we briefly explain this method and the relevant criteria for trapping the IASWs or DLs to use it in the next section. Generally, for deriving the energy-integral equation corresponding to the arbitrary amplitude solitary waves, we may consider the problem in the reference frame of the wave, by using the Galilean transformation as $\xi =x-Vt$, where *V* is the wave speed, and $\xi$ is the common variable in the commoving frame. In terms of the mentioned normalized variables, the Galilean transformation is $\xi ^{'}=x^{'}-{\mathcal {M}}_{\gamma ,e}t^{'}$, where $\xi ^{'}=\frac{\xi }{\lambda _{D,\gamma _{e}}}$, and ${\mathcal {M}}_{\gamma _{e}}=\frac{V}{c_{s,\gamma _{e}}}$ is the adiabatic Mach number, i.e., the fractional wave speed to the generalized ion-sound speed. The adiabatic Mach number is related to the ordinary Mach number as ${\mathcal {M}}_{\gamma _{e}}=\frac{{\mathcal {M}}_{\infty }}{\sqrt{\gamma _{e}}}$, where ${\mathcal {M}}_{\infty }=\frac{V}{c_{s,\infty }}$ is the isothermal Mach number. Then, we may rewrite the transformed equations in terms of the common (normalized) variable $\xi ^{'}$ for deriving the evolution equations of the parameters $n^{'},v^{'},\phi ^{'}$, when the boundary conditions at infinity are $n^{'}\rightarrow 1$, $v^{'}\rightarrow 0$ and $\phi ^{'}\rightarrow 0$ at $|\xi ^{'}|\rightarrow \infty$.

Simultaneously solving the evolution equations by considering the conditions for having a localized solitary wave as $\phi ^{'} , \frac{d\phi ^{'}}{d \xi ^{'}} , \frac{d^{2}\phi ^{'}}{d {\xi ^{'}}^{2}}\rightarrow 0$ when $|\xi ^{'}|\rightarrow \infty$, we may find an energy-integral equation for trapping the IASWs as follows

7 $$\begin{aligned} \frac{1}{2} \left( \frac{d \phi ^{'}}{d \xi ^{'}}\right) ^{2} + \psi (\phi ^{'}, {\mathcal {M}}_{\gamma _{e}};\gamma _{e},Z_{i},\sigma _{ie})=0, \end{aligned}$$

where $\psi (\phi ^{'}, {\mathcal {M}}_{\gamma _{e}};\gamma _{e},Z_{i},\sigma _{ie})$ is Sagdeev’s pseudo-potential function of the plasma, as we will derive it for two cases of cold-ion plasma and warm-ion plasma with a finite temperature.

We may examine the trapping IASWs or possible DLs by using the relevant energy-integral equation as discussed in Ref.^41^, where the necessary conditions are as

(i): $\psi (\phi ^{'}, {\mathcal {M}}_{\gamma _{e}};\gamma _{e},Z_{i},\sigma _{ie})\mid _{\phi ^{'}=0}=0$ (trivial root of pseudo-potential at $\phi ^{'}=0$);
(ii): $\frac{\partial \psi (\phi ^{'}, {\mathcal {M}}_{\gamma _{e}};\gamma _{e},Z_{i},\sigma _{ie})}{\partial \phi ^{'}}\mid _{\phi ^{'}=0}=0$ (the quasi-neutrality condition of plasma);
(iii): $\frac{\partial ^{2}\psi (\phi ^{'}, {\mathcal {M}}_{\gamma _{e}};\gamma _{e},Z_{i},\sigma _{ie})}{\partial {\phi ^{'}}^{2}}\mid _{\phi ^{'}=0}<0$ (the threshold of adiabatic Mach number);
(iv): $\psi (\phi ^{'}_{max},{\mathcal {M}}_{\gamma _{e}};\gamma _{e},Z_{i},\sigma _{ie})=0$ ($\phi ^{'}_{max}$ is the nontrivial root of $\psi$, where $\psi (\phi ^{'}, {\mathcal {M}}_{\gamma _{e}};\gamma _{e},Z_{i},\sigma _{ie})<0$ in the interval $0<|\phi ^{'}|<|\phi ^{'}_{max}|$).

In the latter condition, $\phi ^{'}_{max}$ stands for the absolute maximum of the potential well (maximum amplitude of soliton or possible double layer), which has a central significance for finding the Mach number domains. The maximum potential $\phi ^{'}_{max}$ is the intersection of the pseudo-potential function $\psi (\phi ^{'}, {\mathcal {M}}_{\gamma _{e}};\gamma _{e},Z_{i},\sigma _{ie})$ with the $\phi ^{'}$ axis for a given Mach number and the other parameters, as the condition (iv) confirms it.

Furthermore, the sufficient conditions for trapping the solitons or possible DLs are as:

(v): $\frac{\partial \psi (\phi ^{'}, {\mathcal {M}}_{\gamma _{e}};\gamma _{e},Z_{i},\sigma _{ie})}{\partial \phi ^{'}}\mid _{\phi ^{'}=\phi ^{'}_{max}}\gtrless 0$, which satisfies the positive (negative) slope of the pseudo-potential at $\phi ^{'}_{max}\gtrless 0$ for trapping the compressive (ratefactice) solitary waves;
(vi): $\frac{\partial \psi (\phi ^{'}, {\mathcal {M}}_{\gamma _{e}};\gamma _{e},Z_{i},\sigma _{ie})}{\partial \phi ^{'}}\mid _{\phi ^{'}=\phi ^{'}_{max}}=0$, and $\frac{\partial ^{2}\psi (\phi ^{'}, {\mathcal {M}}_{\gamma _{e}};\gamma _{e},Z_{i},\sigma _{ie})}{\partial {\phi ^{'}}^{2}}\mid _{\phi ^{'}=\phi ^{'}_{max}}<0$, which indicate to the existence of a local maximum at $\phi ^{'}_{max}\gtrless 0$ and the possibility of a typical double layer.

For determining the allowed domains of IASWs, we have to find the situation in which all the criteria (i)-(iv) (necessary conditions) are satisfied simultaneously together with the condition for the reality of the ions number density, by solving the evolution equation of the continuity and momentum transfer equations^41^. Finally, we may analyze the formation condition of the solitary waves, by imposing criterion (v), or the formation condition of the possible DLs, by setting criterion (vi).

## Results and discussion

### Cold-ion plasma

At first, we consider the cold ions regime, where $T_{i} \ll T_{e}$ ($\sigma _{ie}=0$), as it confirms the suitable excitation of the ion oscillations in the plasma. Then, by integrating the transformed equations and considering the boundary conditions at infinity, we have the following equations

8a $$\begin{aligned} n^{'}=\frac{1}{\sqrt{1-\frac{2\phi ^{'}}{\gamma _{e} {\mathcal {M}}_{\gamma _{e}}^{2} }}}, \end{aligned}$$

8b $$\begin{aligned} \frac{d^{2}\phi ^{'}}{d {\xi ^{'}}^{2}}= \gamma _{e} \left[ \left( 1-\frac{1-\gamma _{e}}{\gamma _{e}} \phi ^{'} \right) ^{\frac{1}{\gamma _{e}-1}}-n^{'} \right] . \end{aligned}$$

Note that the first equation is derived by simultaneously solving the continuity and momentum transfer equations in the commoving frame of the wave. It implies a critical potential for establishing the reality of the number density for the ions in the compression/rarefaction of the ion waves, as $\phi ^{'}_{cr}=\frac{\gamma _{e} {\mathcal {M}}_{\gamma _{e}}^{2} }{2}$, where $\phi ^{'}<\phi ^{'}_{cr}$. Note that the reality of the ion number density is disturbed beyond the critical potential (the infinite compression limit), and the propagation of solitary waves is impossible.

Multiplying the Poisson Eq. (8b) by $\frac{d\phi ^{'}}{d {\xi ^{'}}}$, integrating the resultant, and considering the mentioned conditions for having localized solitary waves, we may find the energy-integral equation for trapping the IASWs with the Sagdeev’s pseudo-potential function as follows

9 $$\begin{aligned} \psi (\phi ^{'}, {\mathcal {M}}_{\gamma _{e}};\gamma _{e})= \gamma _{e} \left[ 1- \left( 1-\frac{1-\gamma _{e}}{\gamma _{e}} \phi ^{'} \right) ^{\frac{\gamma _{e}}{\gamma _{e}-1}} \right] +(\gamma _{e} {\mathcal {M}}_{\gamma _{e}} )^{2} \left[ 1- \sqrt{1-\frac{2\phi ^{'}}{\gamma _{e} {\mathcal {M}}_{\gamma _{e}}^{2} }} \right] . \end{aligned}$$

**Figure 1:**
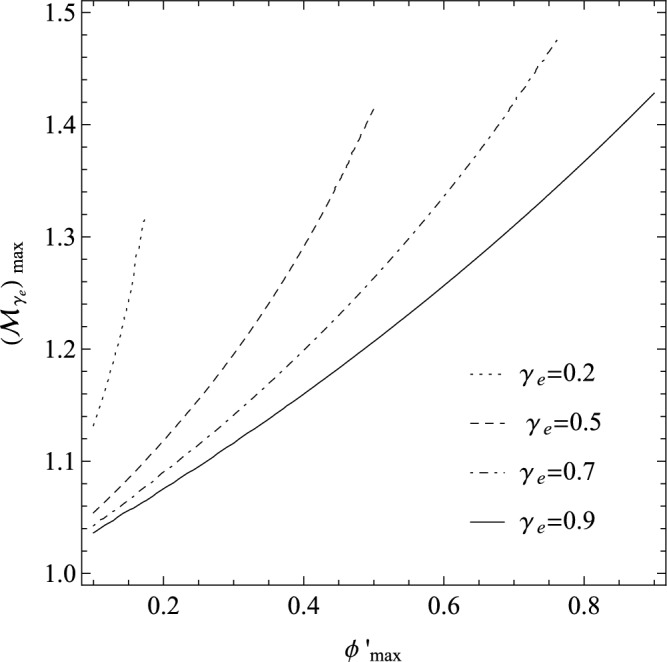
The variation $({\mathcal {M}}_{\gamma _{e}})_{max}$ in terms of $\phi ^{'}_{max}$ for some typical adiabatic indices (the cold-ion limit).

Note that the two conditions (i) and (ii) are spontaneously satisfied for Sagdeev’s pseudo-potential given by Eq. (9). Condition (iii) confirms the minimum energy of the ions for possible excitation of the solitary waves, corresponding to the threshold Mach number. Imposing this condition to the Eq. (9), we may find $({\mathcal {M}}_{\gamma _{e}})_{min}=1$ for a cold plasma, where it is independent of $\phi$ and $\gamma _{e}$. We may also consider the condition (iv) for analyzing the variation $({\mathcal {M}}_{\gamma _{e}})_{max}$ in terms of the maximum potential $\phi ^{'}_{max}$, as we have depicted it in Fig. 1 for some typical adiabatic indices as $\gamma _{e}=0.2,0.5,0.7,0.9$. As we anticipate from the nonlinear plasma physics, it shows that the maximum amplitude of the soliton, i.e. $\phi ^{'}_{max}$, increases with the soliton speed. Moreover, it shows that for a fixed Mach number, the maximum amplitude of the soliton increases with $\gamma _{e}$, i.e., we have the IASWs with higher amplitudes towards the equilibrium states.

**Figure 2:**
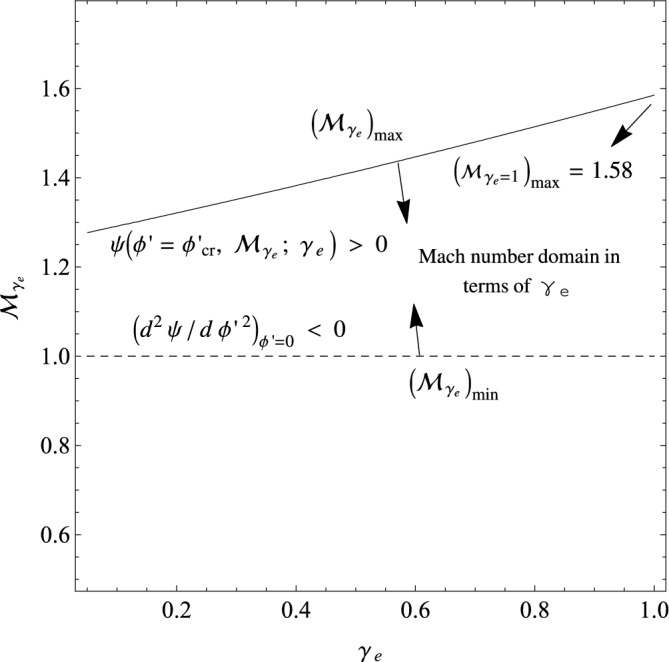
The allowed domains of $(\gamma _{e},{\mathcal {M}}_{\gamma _{e}})$ for the IASWs (the cold-ion limit).

In one viewpoint for showing the interval of the Mach number, we may consider $\phi ^{'}_{cr}=\frac{\gamma _{e} {\mathcal {M}}_{\gamma _{e}}^{2} }{2}$ as the maximum width of the possible potential wells, i.e., the maximum possible for the potential, and then by solving the inequality $\psi (\phi ^{'}=\phi ^{'}_{cr}, {\mathcal {M}}_{\gamma _{e}};\gamma _{e})>0$ (the necessary condition for forming a potential well), we can extract the maximum possible for the adiabatic Mach number as a function of $\gamma _{e}$. Then, we may display the interval of the adiabatic Mach number as $({\mathcal {M}}_{\gamma _{e}})_{min}<{\mathcal {M}}_{\gamma _{e}}<({\mathcal {M}}_{\gamma _{e}})_{max}$ in the plane of ${\mathcal {M}}_{\gamma _{e}}$ versus $\gamma _{e}$, as we have depicted it in Fig. 2. Here, the regions between $({\mathcal {M}}_{\gamma _{e}})_{min}$ and $({\mathcal {M}}_{\gamma _{e}})_{max}$ correspond to the allowed Mach number domains in the $(\gamma _{e},{\mathcal {M}}_{\gamma _{e}})$ plane. As we see from Fig. 2, in the isothermal limit $\gamma _{e}\rightarrow 1$, the classical Mach number domains for a Maxwellian cold plasma may be recovered as $1<{\mathcal {M}}<1.58$^40,44^.

**Figure 3:**
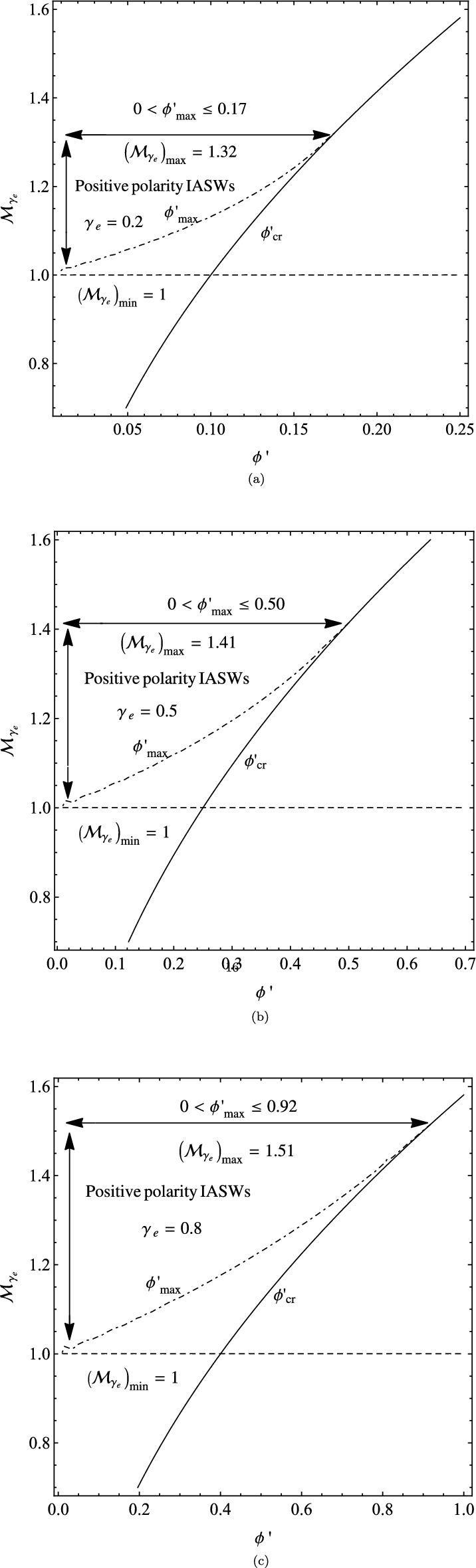
The allowed $(\phi ^{'},{\mathcal {M}}_{\gamma _{e}})$ domains for trapping the positive polarity IASWs in the cold-ions plasma: (**a**) when $\gamma _{e}=0.2$, in which $1<{\mathcal {M}}_{\gamma _{e}}<1.32$ and $0<\phi ^{'}_{max}<0.17$; (**b**) when $\gamma _{e}=0.5$, in which $1<{\mathcal {M}}_{\gamma _{e}}<1.41$ and $0<\phi ^{'}_{max}<0.50$; and (**c**) when $\gamma _{e}=0.8$, in which $1<{\mathcal {M}}_{\gamma _{e}}<1.51$ and $0<\phi ^{'}_{max}<0.92$.

Another approach for extracting the solutions of the energy-integral equations is analyzing the allowed $(\phi ^{'},{\mathcal {M}}_{\gamma _{e}})$ domains for trapping the IASWs^41^. In three panels of Fig. 3, we have displayed the overlap of three constraints (iii),(iv) and $\phi ^{'}<\phi ^{'}_{cr}$ in the $(\phi ^{'},{\mathcal {M}}_{\gamma _{e}})$ planes, where the panels (a), (b) and (c) correspond respectively to the plasma with adiabatic indices $\gamma _{e}=0.2$, $\gamma _{e}=0.5$ and $\gamma _{e}=0.8$. The horizontal dashed line of these figures indicates the threshold Mach number value, $({\mathcal {M}}_{\gamma _{e}})_{min}=1$, where the upper areas of this line correspond to the condition $\frac{\partial ^{2}\psi (\phi ^{'}, {\mathcal {M}}_{\gamma _{e}};\gamma _{e})}{\partial {\phi ^{'}}^{2}}\mid _{\phi ^{'}=0}<0$, which confirms the existence of a local maximum for the pseudo-potential $\psi (\phi ^{'}, {\mathcal {M}}_{\gamma _{e}};\gamma _{e})$ at zero potential. The left areas of the solid curve satisfy the reality of the ion number density as $\phi ^{'}<\phi ^{'}_{cr}=\frac{\gamma _{e} {\mathcal {M}}_{\gamma _{e}}^{2} }{2}$. Furthermore, the dot-dashed curve represents the nonzero roots of the pseudo-potential as $\psi (\phi ^{'}_{max},{\mathcal {M}}_{\gamma _{e}};\gamma _{e})=0$, where only the left regions of this curve satisfy the negativity of the pseudo-potential as $\psi (\phi ^{'}, {\mathcal {M}}_{\gamma _{e}};\gamma _{e})<0$. The dot-dashed and the solid curves converge to the intersection point $({\phi ^{'}}^{\star }_{max},({\mathcal {M}}_{\gamma _{e}})_{max})$, beyond which the propagation of solitary waves is impossible. The maximum possibilities for the potential and the adiabatic Mach numbers, i.e., $({\phi ^{'}}^{\star }_{max},({\mathcal {M}}_{\gamma _{e}})_{max})$, for three panels (a), (b), and (c) are respectively (0.17, 1.32) when $\gamma _{e}=0.2$, (0.50, 1.41) when $\gamma _{e}=0.5$, and (0.92, 1.51) when $\gamma _{e}=0.8$.

As we see from all panels of Fig. 3, the slope of the dot-dashed curve is always positive, i.e.,

$\frac{\partial \psi (\phi ^{'}, {\mathcal {M}}_{\gamma _{e}};\gamma _{e})}{\partial \phi ^{'}}\mid _{\phi ^{'}=\phi ^{'}_{max}}>0$, and so $\psi (\phi ^{'}, {\mathcal {M}}_{\gamma _{e}};\gamma _{e})$ has no local maximum for $\phi ^{'}_{max}>0$. It shows that only positive polarity IASWs are possible (compressive solitons), and no DLs are possible in the plasma. We have displayed the $(\phi ^{'},{\mathcal {M}}_{\gamma _{e}})$ domains for trapping the IASWs in the kappa-distributed cold plasma in three panels of Fig. 3. The allowed adiabatic Mach number domains for the positive polarity IASWs are written as: $1<{\mathcal {M}}_{\gamma _{e}}<1.32$ with the soliton amplitudes as $0<\phi ^{'}_{max}<0.17$ when $\gamma _{e}=0.2$; $1<{\mathcal {M}}_{\gamma _{e}}<1.41$ with the soliton amplitudes as $0<\phi ^{'}_{max}<0.50$ when $\gamma _{e}=0.5$; and $1<{\mathcal {M}}_{\gamma _{e}}<1.51$ with the soliton amplitudes as $0<\phi ^{'}_{max}<0.92$ when $\gamma _{e}=0.8$. We see that the allowed $(\phi ^{'},{\mathcal {M}}_{\gamma _{e}})$ domains extend towards the isothermal limit $\gamma _{e}\rightarrow 1$. A typical adiabatic Mach number in the allowed regions of $({\mathcal {M}}_{\gamma _{e}})_{min}<{\mathcal {M}}_{\gamma _{e}}<({\mathcal {M}}_{\gamma _{e}})_{max}$ corresponds to a potential well in the related area $0<\phi ^{'}<\phi ^{'}_{max}$, where $\phi ^{'}_{max}$ is the relevant soliton amplitude.

**Figure 4:**
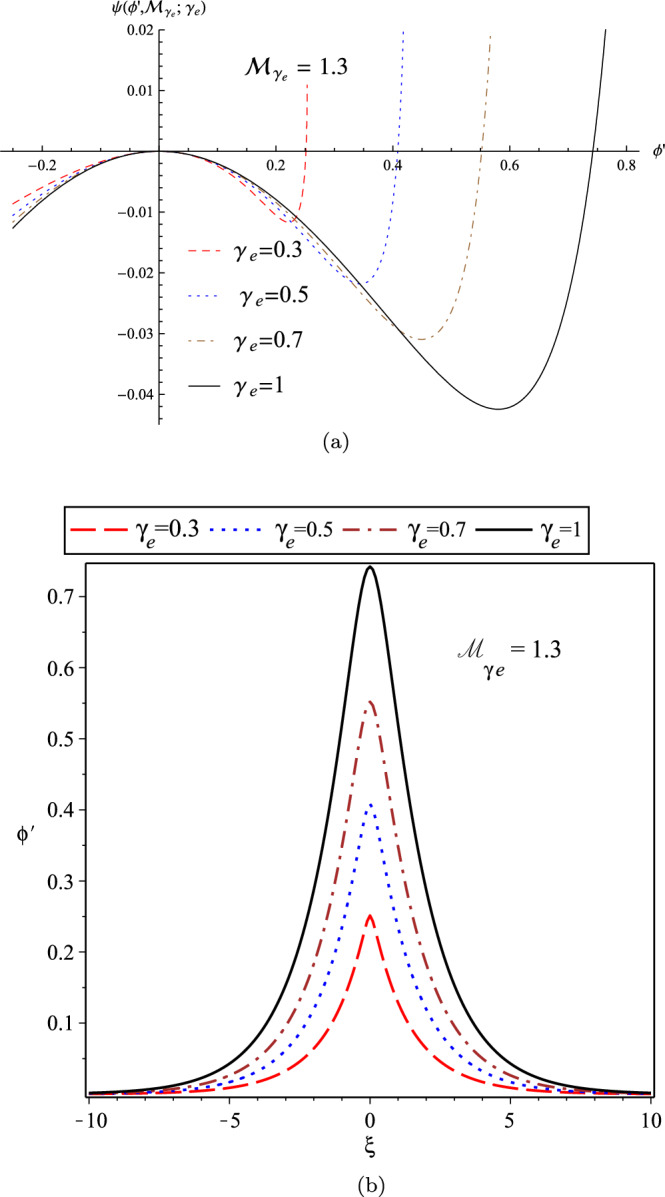
(**a**) The variation of Sagdeev’s pseudo-potential function; and (**b**) The variation of soliton profile; for the cold-ions plasma in terms of $\gamma _{e}$ for the fixed adiabatic Mach number ${\mathcal {M}}_{\gamma _{e}}=1.3$.

In Fig. 4, we have plotted the variation of Sagdeev’s pseudo-potential function in terms of $\gamma _{e}$ for a fixed adiabatic Mach number as ${\mathcal {M}}_{\gamma _{e}}=1.3$ (panel (a)), and also the related soliton profiles by numerically solving the energy-integral equation $\frac{1}{2}(\frac{d \phi ^{'}}{d \xi ^{'}})^{2}+\psi (\phi ^{'}, {\mathcal {M}}_{\gamma _{e}};\gamma _{e})=0$ (panel (b)). The width and amplitude of the IASWs increase with $\gamma _{e}$.

**Figure 5:**
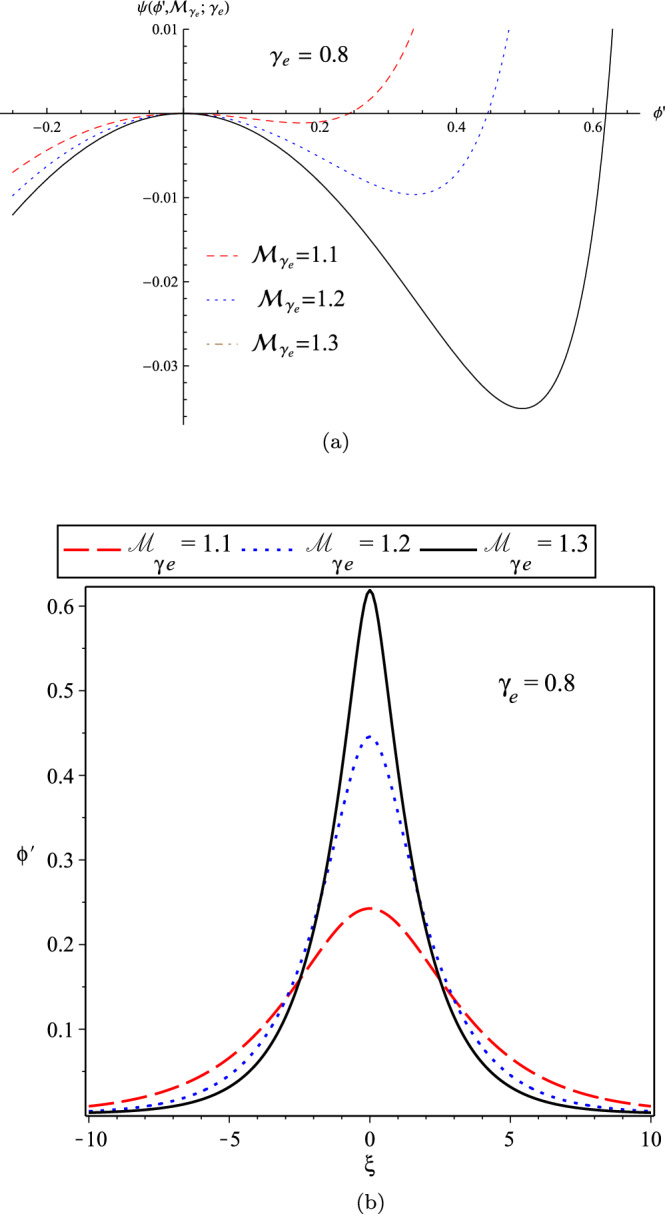
(**a**) The variation of Sagdeev’s pseudo-potential function; and (**b**) The variation of soliton profile; for the cold-ions plasma in terms of ${\mathcal {M}}_{\gamma _{e}}$ for the fixed polytropic index $\gamma _{e}=0.8$.

Furthermore, in panel (a) of Fig. 5, we have plotted the variation of the pseudo-potential function in terms of ${\mathcal {M}}_{\gamma _{e}}$ for a fixed polytropic index as $\gamma _{e}=0.8$, and also the related soliton profiles as depicted in the panel (b). We see that the solitary wave profile becomes sharper for the solitons with higher speeds, where ${\mathcal {M}}_{\gamma _{e}}$ increases.

### Warm-ion plasma with finite temperature

For a warm plasma with finite temperature ions, by integrating the transformed continuity and momentum transfer equations (in terms of $\xi ^{'}$) and imposing the boundary conditions at infinity, we may find the following multi-dimensional equation in terms of $n^{'}$

10 $$\begin{aligned} \left( \frac{2\sigma _{ie}}{Z_{i}\gamma _{e}} \cdot \frac{\gamma _{i}}{\gamma _{i}-1} \right) {n^{'}}^{\gamma _{i}+1} +\left( {\mathcal {M}}_{\gamma _{e}}^{2}-\frac{2\phi ^{'}}{\gamma _{e}} +\frac{2\sigma _{ie}}{Z_{i}\gamma _{e}} \cdot \frac{\gamma _{i}}{\gamma _{i}-1}\right) {n^{'}}^{2} +{\mathcal {M}}_{\gamma _{e}}^{2}=0. \end{aligned}$$

For finding an explicit Sagdeev’s pseudo-potential function, we consider the adiabatic ions in one dimension compression/rarefaction, where $d_{i}=1$ and $\gamma _{i}=3$, then the Eq. (10) becomes a fourth order equation in terms of $n^{'}$ as follows

11 $$\begin{aligned} \frac{3\sigma _{ie}}{Z_{i}\gamma _{e}} {n^{'}}^{4}- \left( {\mathcal {M}}_{\gamma _{e}}^{2}-\frac{2\phi ^{'}}{\gamma _{e}}+\frac{3\sigma _{ie}}{Z_{i}\gamma _{e}}\right) {n^{'}}^{2} +{\mathcal {M}}_{\gamma _{e}}^{2}=0, \end{aligned}$$

where it has two (acceptable) positive solutions as follows

12 $$\begin{aligned} n_{\pm }^{'}=\frac{1}{\sqrt{\frac{6\sigma _{ie}}{Z_{i}\gamma _{e}}}} \left\{ {\mathcal {M}}_{\gamma _{e}}^{2}+ \frac{3\sigma _{ie}}{Z_{i}\gamma _{e}} -\frac{2\phi ^{'}}{\gamma _{e}} \pm \sqrt{\left( {\mathcal {M}}_{\gamma _{e}}^{2}+ \frac{3\sigma _{ie}}{Z_{i}\gamma _{e}} -\frac{2\phi ^{'}}{\gamma _{e}} \right) ^{2} -\frac{12\sigma _{ie}}{Z_{i}\gamma _{e}} {\mathcal {M}}_{\gamma _{e}}^{2}} \right\} ^{\frac{1}{2}}. \end{aligned}$$

For determining the acceptable branch of $n_{\pm }^{'}$, the following conditions have to be considered

- The positivity of $n^{'}$,
- The reality of $n^{'}$,
- $n^{'}$ has to tend smoothly to $n^{'}\rightarrow 1$, at the equilibrium state $\phi ^{'}\rightarrow 0$,
- $n^{'}$ has to tend smoothly to $n^{'}\rightarrow \frac{1}{\sqrt{1-\frac{2\phi ^{'}}{\gamma _{e} {\mathcal {M}}_{\gamma _{e}}^{2} }}}$, at the cold ions limit $\sigma _{ie}\rightarrow 0$.

Considering these conditions shows that only $n_{-}^{'}$ satisfies the correct cold ions limit at $\sigma _{ie}\rightarrow 0$, where its reality gives a critical potential as

13 $$\begin{aligned} \phi _{cr}^{+}=\frac{1}{2} \left( \sqrt{\gamma _{e}} {\mathcal {M}}_{\gamma _{e}}-\sqrt{\frac{3\sigma _{ie}}{Z_{i}}}\right) ^{2}. \end{aligned}$$

We may derive the critical potential by considering the positivity of the expression under the square root symbol in Eq. (12), where we have rearranged it as the completed square. Note that we have real solutions for the potentials in the range $0\le \phi ^{'}<\phi _{cr}^{+}$. Furthermore, $n_{-}^{'}$ satisfies the proper equilibrium limit at $\phi ^{'}\rightarrow 0$, when the adiabatic Mach number takes the following values

14 $$\begin{aligned} {\mathcal {M}}_{\gamma _{e}}> \sqrt{\frac{3\sigma _{ie}}{Z_{i}\gamma _{e}}}. \end{aligned}$$

The transformed Poisson equation for this case is the same as Eq. (8b) with $n_{-}^{'}$ instead of $n^{'}$. Inserting $n_{-}^{'}$ to the Poisson equation, multiplying it by $\frac{d\phi ^{'}}{d {\xi ^{'}}}$, and integrating by considering the boundary conditions for having a localized solitary wave, we may find the following Sagdeev’s pseudo-potential function

15 $$\begin{aligned}{} & {} \psi (\phi ^{'}, {\mathcal {M}}_{\gamma _{e}};\gamma _{e},Z_{i},\sigma _{ie})= \gamma _{e} \left[ 1- \left( 1-\frac{1-\gamma _{e}}{\gamma _{e}} \phi ^{'} \right) ^{\frac{\gamma _{e}}{\gamma _{e}-1}} \right] \nonumber \\{} & {} -\gamma _{e}^{2} \left( \frac{3\sigma _{ie}}{Z_{i}\gamma _{e}} {\mathcal {M}}_{\gamma _{e}}^{6} \right) ^{\frac{1}{4}} \left[ (\Theta ^{\frac{1}{2}}-\Theta _{0}^{\frac{1}{2}}) +\frac{1}{3}(\Theta ^{-\frac{3}{2}}-\Theta _{0}^{-\frac{3}{2}}) \right] , \end{aligned}$$

where $\Theta$ is defined as follows

16 $$\begin{aligned} \Theta =\frac{{\mathcal {M}}_{\gamma _{e}}^{2}+ \frac{3\sigma _{ie}}{Z_{i}\gamma _{e}} -\frac{2\phi ^{'}}{\gamma _{e}}}{\sqrt{\frac{12\sigma _{ie}}{Z_{i}\gamma _{e}}} {\mathcal {M}}_{\gamma _{e}}} + \sqrt{\frac{\left( {\mathcal {M}}_{\gamma _{e}}^{2}+ \frac{3\sigma _{ie}}{Z_{i}\gamma _{e}} -\frac{2\phi ^{'}}{\gamma _{e}}\right) ^{2}}{\frac{12\sigma _{ie}}{Z_{i}\gamma _{e}} {\mathcal {M}}_{\gamma _{e}}^{2}}-1}, \end{aligned}$$

and $\Theta _{0}=\Theta (\phi ^{'}=0)$. The method for deriving the energy-integral equation and Sagdeev’s pseudo-potential function in the case of warm plasma exists in the Supplementary Material.

**Figure 6:**
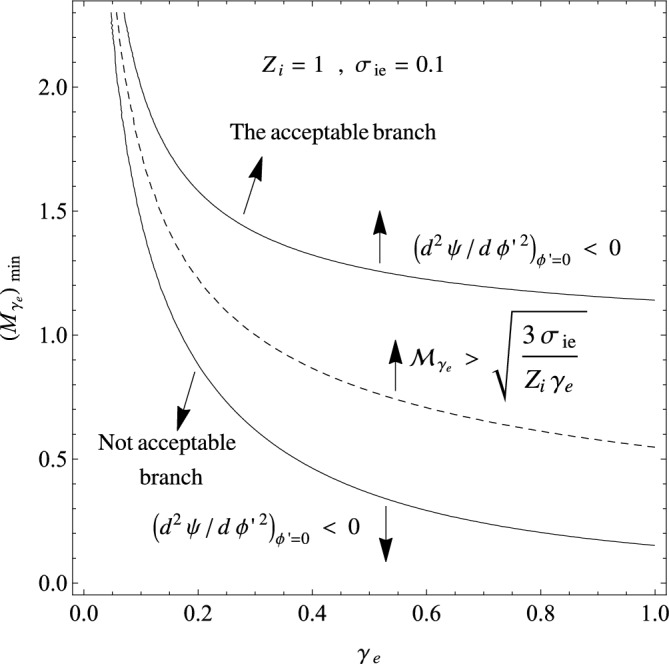
The variation $({\mathcal {M}}_{\gamma _{e}})_{min}$ in warm plasma in terms of $\gamma _{e}$ for the fixed parameters $Z_{i}=1$ and $\sigma _{ie}=0.1$. The upper branch is acceptable, where the true equilibrium limit at $\phi ^{'}\rightarrow 0$ is satisfied.

As we mentioned, the minimum energy of the ions for possible excitation of the solitary waves corresponds to the threshold Mach number. It is given by using the relation $\frac{\partial ^{2}\psi (\phi ^{'}, {\mathcal {M}}_{\gamma _{e}};\gamma _{e},Z_{i},\sigma _{ie})}{\partial {\phi ^{'}}^{2}}\mid _{\phi ^{'}=0}=0$. In the cold-ion plasma limit, this condition led to $({\mathcal {M}}_{\gamma _{e}})_{min}=1$. However, for the warm ion plasma, imposing this condition yields a criterion as a function of the other parameters, i.e., $\gamma _{e},Z_{i}$ and $\sigma _{ie}$. In Fig. 6, we have depicted this condition for the fixed parameters $Z_{i}=1$ and $\sigma _{ie}=0.1$, which indicates that the variation of $({\mathcal {M}}_{\gamma _{e}})_{min}$ in terms of $\gamma _{e}$ has two branches. In this figure, we have also depicted the allowed domains for satisfying the proper equilibrium limit at $\phi ^{'}\rightarrow 0$, as formulated in the relation (14). Figure 6 shows that only the upper branch is acceptable, denoting the valid variation of $({\mathcal {M}}_{\gamma _{e}})_{min}$ in terms of $\gamma _{e}$. It shows the threshold of Mach number decreases by increasing the polytropic index $\gamma$.

**Figure 7:**
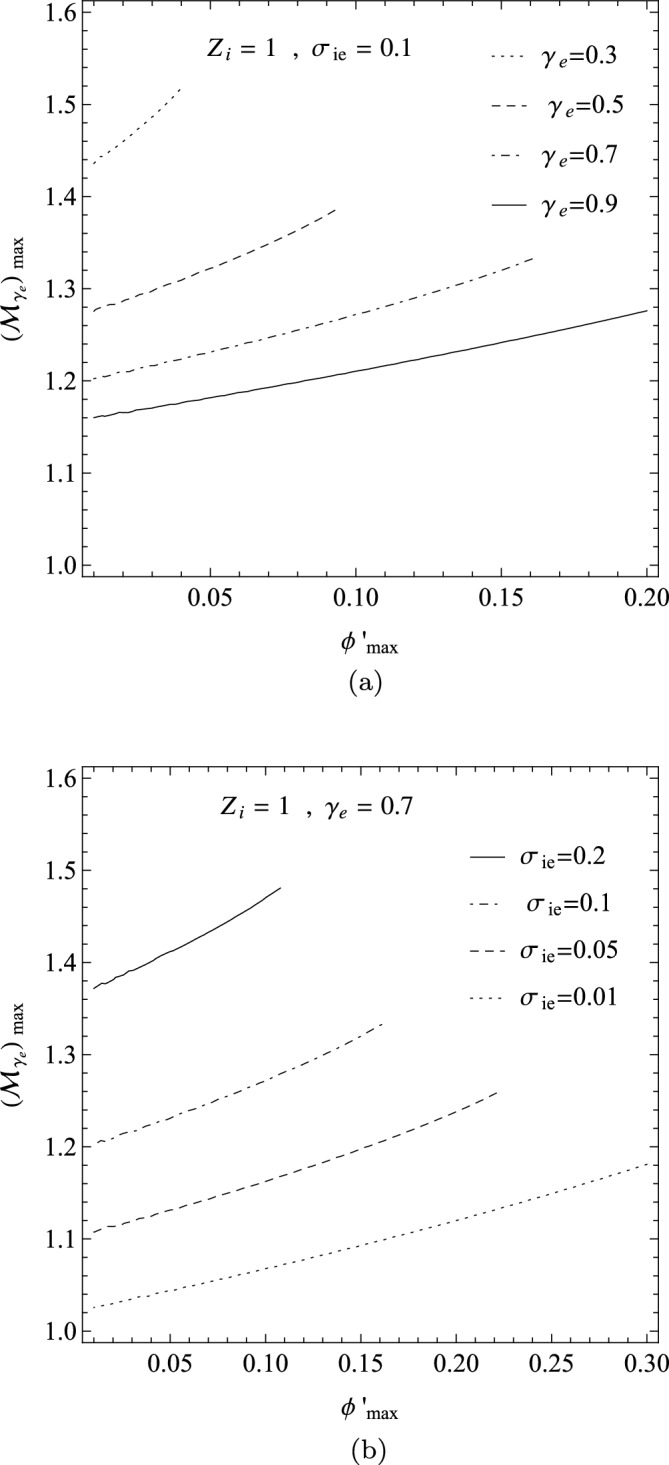
The variation $({\mathcal {M}}_{\gamma _{e}})_{max}$ for a warm plasma in terms of $\phi ^{'}_{max}$: (**a**) for some adiabatic indices and the fixed parameters $Z_{i}=1$ and $\sigma _{ie}=0.1$; (**b**) for some fractional ion to electron temperatures and the fixed parameters $Z_{i}=1$ and $\gamma _{e}=0.7$.

We may extend the analysis discussed in the prior section to Sagdeev’s pseudo-potential function for the warm plasma as follows. The panel (a) in Fig. 7 shows the variation $({\mathcal {M}}_{\gamma _{e}})_{max}$ in terms of the maximum potential $\phi ^{'}_{max}$ for some adiabatic indices as $\gamma _{e}=0.3,0.5,0.7,0.9$ and for the fixed parameters $Z_{i}=1$ and $\sigma _{ie}=0.1$, while the panel (b) is depicted for some fractional ion to electron temperatures as $\sigma _{ie}=0.01,0.05,0.1,0.2$ and for the fixed parameters $Z_{i}=1$ and $\gamma _{e}=0.7$. Fig. 7) confirms the result of the prior section. It also shows that for a fixed Mach number, the maximum amplitude of the soliton decreases with $\sigma _{ie}$, i.e., we have the IASWs with smaller amplitudes in the plasmas with warmer ions.

**Figure 8:**
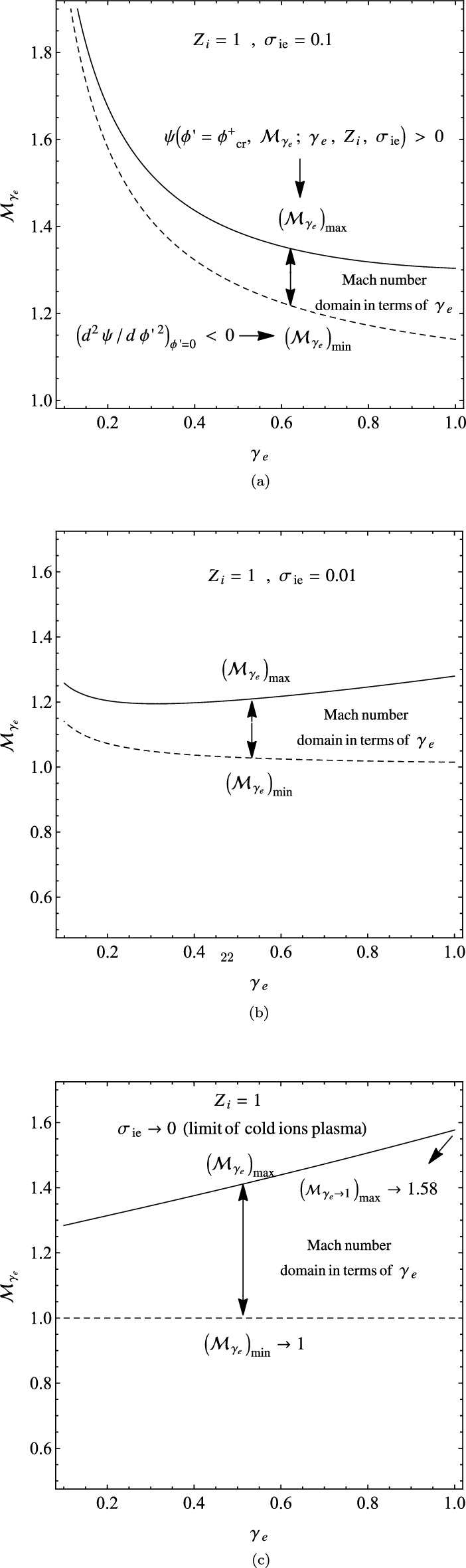
The variation of the allowed $(\gamma _{e},{\mathcal {M}}_{\gamma _{e}})$ domains in a warm plasma, when $Z_{i}=1$ and (**a**) $\sigma _{ie}=0.1$; (**b**) $\sigma _{ie}=0.01$; and (c) $\sigma _{ie}\rightarrow 0$ (the cold-ion limit).

In three panels of Fig. 8, we have depicted the domains of adiabatic Mach number ${\mathcal {M}}_{\gamma _{e}}$ in terms of $\gamma _{e}$, where the panels (a),(b) and (c) correspond respectively to the case with $\sigma _{ie}=0.1$, $\sigma _{ie}=0.01$ and the asymptotic limit $\sigma _{ie}\rightarrow 0$, where $Z_{i}=1$ for all of them. The regions between $({\mathcal {M}}_{\gamma _{e}})_{min}$ and $({\mathcal {M}}_{\gamma _{e}})_{max}$ correspond to the allowed Mach number domains in the $(\gamma _{e},{\mathcal {M}}_{\gamma _{e}})$ plane. Figure 8 shows that the allowed domains of the IASWs shrink with the temperature of the plasma ions. Significantly, the panels (c) of Fig. 8 at the cold-ion limit $\sigma _{ie}\rightarrow 0$ is in agreement with the result of cold plasma model, as given in Fig. 2. Furthermore, the allowed Mach number domain tends smoothly to the classical interval $1<{\mathcal {M}}_{\gamma _{e}}<1.58$ at the asymptotic limits $\gamma _{e} \rightarrow 1$ and $\sigma _{ie}\rightarrow 0$^40^.

**Figure 9:**
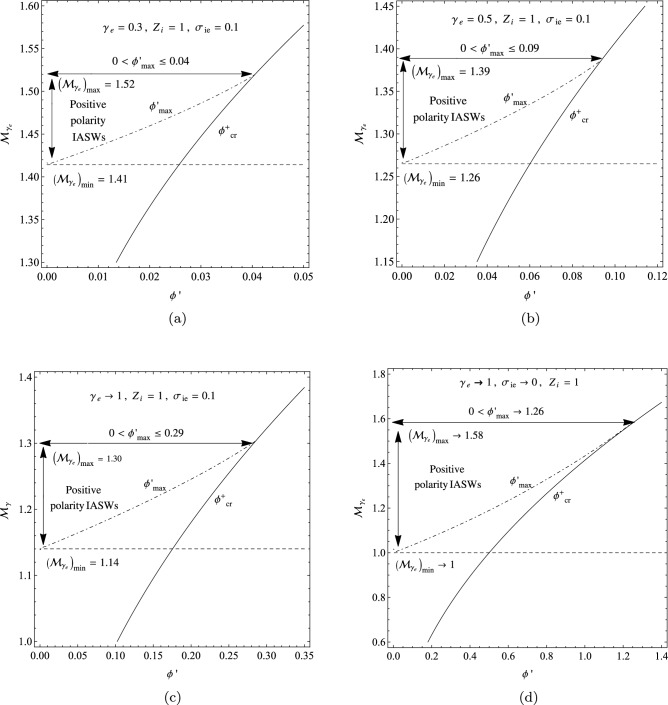
The variation of the allowed $(\phi ^{'},{\mathcal {M}}_{\gamma _{e}})$ domains in a warm plasma with $Z_{i}=1$: (**a**) when $\gamma _{e}=0.3$ and $\sigma _{ie}=0.1$; (**b**) when $\gamma _{e}=0.5$ and $\sigma _{ie}=0.1$; (**c**) when $\gamma _{e}\rightarrow 1$ (the isothermal limit) and $\sigma _{ie}=0.1$; and (**d**) when $\gamma _{e}\rightarrow 1$ and $\sigma _{ie}\rightarrow 0$ (the cold-ion limit).

In four panels of Fig. 9, we have compared the allowed $(\phi ^{'},{\mathcal {M}}_{\gamma _{e}})$ domains by variations of the polytropic index $\gamma _{e}$ and the fractional temperature $\sigma _{ie}$. Here, the panel (a) corresponds to the plasma with $\gamma _{e}=0.3$ and $\sigma _{ie}=0.1$, the panel (b) corresponds to the values $\gamma _{e}=0.5$ and $\sigma _{ie}=0.1$, the panel (c) corresponds to the values $\gamma _{e}\rightarrow 1$ and $\sigma _{ie}=0.1$, and the panel (d) corresponds to the asymptotic limits $\gamma _{e}\rightarrow 1$ and $\sigma _{ie}\rightarrow 0$, where $Z_{i}=1$ for all of them. Figure 9 shows that both $({\mathcal {M}}_{\gamma _{e}})_{min}$ and $({\mathcal {M}}_{\gamma _{e}})_{max}$ decrease with $\gamma _{e}$ towards the equilibrium state at $\gamma _{e}\rightarrow 1$, and also they fall with the temperature of the plasma ions. The panel (d) of Fig. 9 is in agreement with the $(\phi ,{\mathcal {M}})$ domains of cold-ion plasma with isothermal electrons^41^.

Our analysis shows that the maximum intervals of the allowed adiabatic Mach numbers and the allowed potentials for the propagation of IASWs happen at the limit of isothermal electrons ($\gamma _{e}\rightarrow 1$) and in the case of the cold-ion limit ($\sigma _{ie}\rightarrow 0$), where they are given by $1<{\mathcal {M}}_{\gamma _{e}}<1.58$ and $0<\phi ^{'}_{max}<1.26$, as addressed in the classical plasmas^45^.

**Figure 10:**
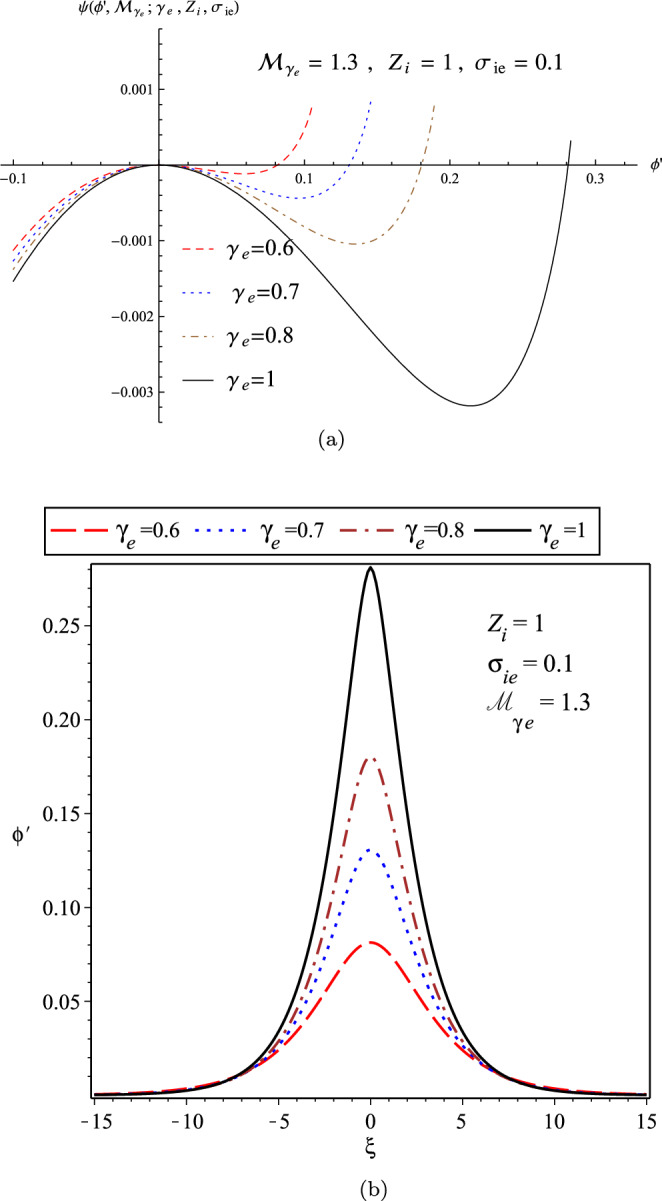
(**a**) The variations of pseudo-potential function; and (**b**) The variations of soliton profile; in terms of $\gamma _{e}$ for a warm plasma when ${\mathcal {M}}_{\gamma _{e}}=1.3$, $\sigma _{ie}=0.1$ and $Z_{i}=1$.

Panel (a) of Fig. 10 shows the variation of pseudo-potential function for the warm plasma in terms of $\gamma _{e}$ for the fixed parameters as ${\mathcal {M}}_{\gamma _{e}}=1.3$, $\sigma _{ie}=0.1$ and $Z_{i}=1$, and the related soliton profiles as given in the panel (b). It confirms again that the width and amplitude of the IASWs increase with $\gamma _{e}$, in agreement with the result derived by the perturbation technique^39^.

**Figure 11:**
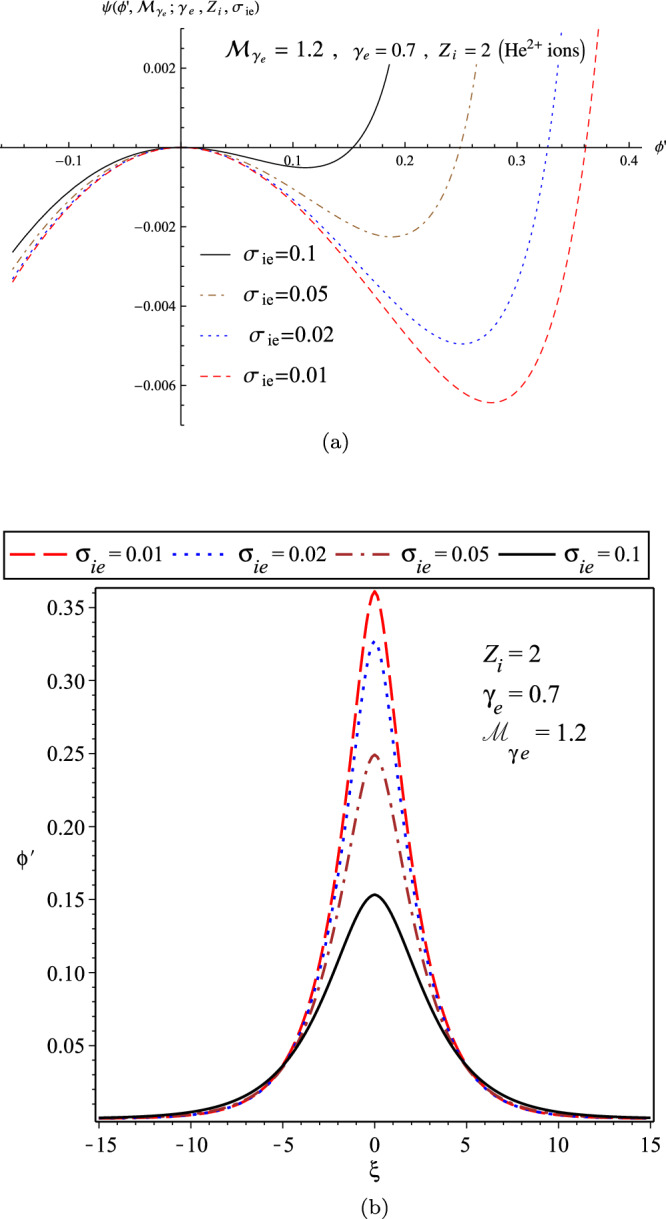
(**a**) The variations of pseudo-potential function; and (**b**) The variations of soliton profile; in terms of $\sigma _{ie}$ for a warm plasma when $\gamma _{e}=0.7$, ${\mathcal {M}}_{\gamma _{e}}=1.2$, and $Z_{i}=2$ (denoting the $He^{2+}$ ions).

Furthermore, in panel (a) of Fig. 11, we have plotted the variation of the pseudo-potential function in terms of $\sigma _{ie}$ for the fixed parameters as $\gamma _{e}=0.7$, ${\mathcal {M}}_{\gamma _{e}}=1.2$, and $Z_{i}=2$ (denoting to the plasma with $He^{2+}$ ions), and also the related soliton profiles as depicted in the panel (b). It shows that the width and amplitude of the IASWs decrease with the temperature of the ions, in agreement with the related result as derived by the perturbation technique^39^.

**Figure 12:**
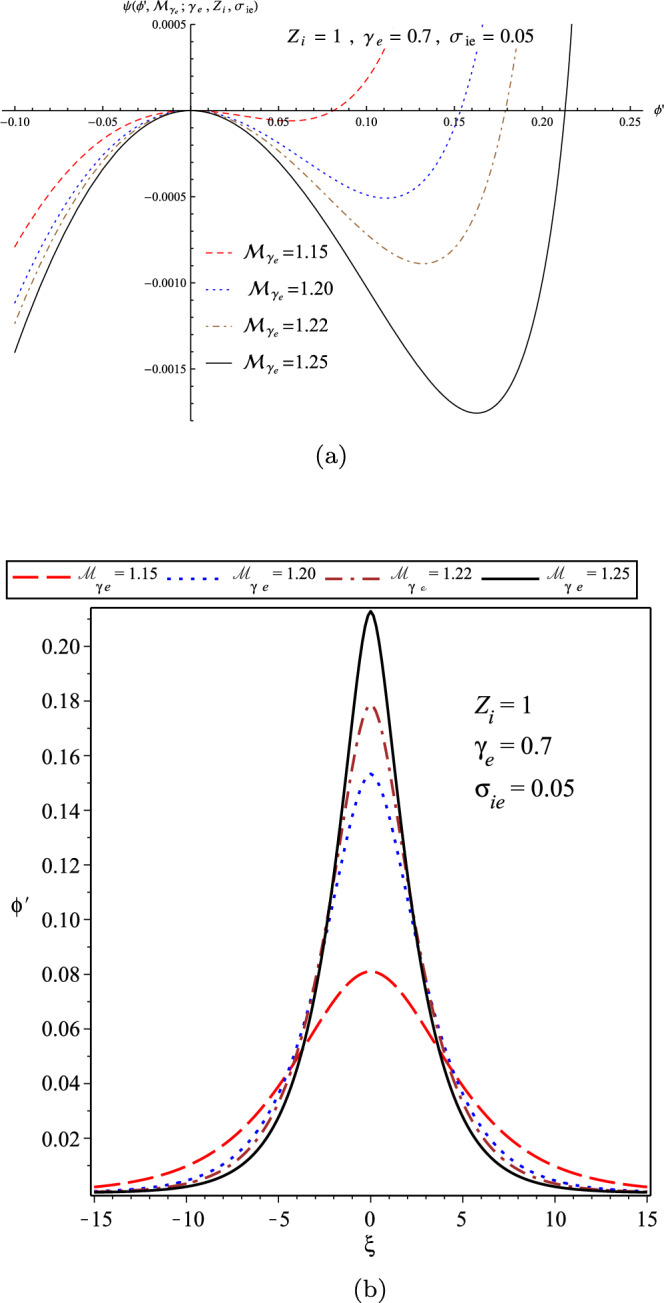
(**a**) The variations of pseudo-potential function; and (**b**) The variations of soliton profile; in terms of ${\mathcal {M}}_{\gamma _{e}}$ for a warm plasma when $\gamma _{e}=0.7$, $\sigma _{ie}=0.05$, and $Z_{i}=1$ (denoting the $H^{+}$ ions).

Finally, panel (a) of Fig. 12 depicts the variation of the pseudo-potential function in terms of ${\mathcal {M}}_{\gamma _{e}}$ for the fixed parameters as $\gamma _{e}=0.7$, $\sigma _{ie}=0.05$, and $Z_{i}=1$ (denoting to the plasma with $H^{+}$ ions), and also the related soliton profiles as plotted in the panel (b). It confirms that with increasing ${\mathcal {M}}_{\gamma _{e}}$, the solitary wave profile becomes sharper, which is in agreement with the similar result as derived by the perturbation technique^39^.

We note that the relevant kappa and polytropic indices used in the numerical analysis of this paper are very close to the reported data in various regions of space physics. We may categorize the observational data into three regions: (i) The far-equilibrium regions, where the related thermodynamic processes are sub-isothermal and the polytropic indices are in the range $\gamma _{e}<1$. For example, the ambient solar wind (SW) regions with $\kappa _{0}\sim 0$^46^, where the polytropic index of kappa distributed particles is very close to the anti-equilibrium state $\gamma _{e}\sim 0$^39^; the outer heliosphere regions with $\kappa _{0}\sim 0.13$^47^ and $\gamma _{e}\sim 0.11$^39^; and the inner heliosheath (IH) regions with $\kappa _{0}\sim 0.25$^12^ and $\gamma _{e}\sim 0.2$^39^. (ii) The regions close to the thermal equilibrium, where the related thermodynamics processes are isothermal, and the polytropic indices are $\gamma _{e}\sim 1$. The hotter and denser space plasmas belong to this case., e.g., the lower solar corona $\mathrm {e^{-}}$ with $\kappa _{0}\sim 15.5$^48^ and $\gamma _{e}\sim 0.94$^39^; the HII $\mathrm {e^{-}}$ regions with $\kappa _{0}\sim 10.5$^49^ and $\gamma _{e}\sim 0.91$^39^; and the planetary nebulae with $\kappa _{0}\sim 100$^50^, where the polytropic index is very close to $\gamma _{e}\sim 1$^39^. (iii) The regions in which thermodynamics processes are close to the escape state, where the transitions between the near/far-equilibrium states may happen. We may refer to the slow solar wind $\mathrm {e^{-}}$ (Ulysses) plasmas with $\kappa _{0}\sim 0.9$^51^, where the polytropic index is given by $\gamma _{e}\sim 0.47$^39^; and the fast solar wind $\mathrm {He^{+}}$ plasmas with $\kappa _{0}\sim 1.15$^52^ and $\gamma _{e}\sim 0.53$^39^.

## Conclusion

In this paper, we studied the propagation and the allowed domains of the IASWs in space plasmas with invariant kappa-distributed electrons and adiabatic ions. We discussed the nonlinear features of the invariant IAWs by deriving the energy-integral equation in Sagdeev’s pseudo-potential approach. The structure of solitary wave solutions was studied in terms of the polytropic index associated with the kappa distributed electrons ($\gamma _{e}$), the adiabatic (extended) Mach number (${\mathcal {M}}_{\gamma _{e}}$), and the fractional ion to electron temperature ($\sigma _{ie}$). The value of the polytropic index varies between $0<\gamma _{e}\le 1$, where lower/higher indices show whether the plasma is far from/close to the equilibrium state. We derived and analyzed Sagdeev’s pseudo-potential function for two cases, i.e., the cold-ion plasma and the warm plasma with finite-temperature ions. The allowed domains of IASWs were presented both in $(\gamma _{e},{\mathcal {M}}_{\gamma _{e}})$ plane and in $(\phi ^{'},{\mathcal {M}}_{\gamma _{e}})$ plane. The summary of our results is as follows:

- As we anticipate from the nonlinear plasma physics, the maximum amplitude of the soliton increases with the soliton speed.
- The width and amplitude of the IASWs increase towards the equilibrium state, while they decrease with the temperature of the ions.
- The solitary wave profile becomes sharper with increasing ${\mathcal {M}}_{\gamma _{e}}$ (for the solitons with more speeds).
- The maximum amplitude of the soliton decreases with the fractional ion to electron temperature, i.e., the IASWs with smaller amplitudes happen in the plasmas with warmer ions.
- In the case of cold-ion plasma, the threshold of Mach number (the minimum energy of the ions for possible excitation of the solitary waves) is independent of $\gamma _{e}$, where it is given by $({\mathcal {M}}_{\gamma _{e}})_{min}=1$. On the other hand, the threshold of Mach number in the case of warm ion plasma is a function of the other parameters, i.e., $\gamma _{e}$, $\sigma _{ie}$, and $Z_{i}$.
- In the cold-ion plasma, the allowed $(\phi ^{'},{\mathcal {M}}_{\gamma _{e}})$ domains is extended towards the isothermal plasma at the limit $\gamma _{e}\rightarrow 1$.
- The temperature ratio is an essential factor for determining the maximum amplitude and allowed domains of the IASWs. Changing the ion temperature significantly modifies the maximum amplitude of the soliton.
- In a warm plasma, both the lower and upper limits of the Mach number, i.e., $({\mathcal {M}}_{\gamma _{e}})_{min}$ and $({\mathcal {M}}_{\gamma _{e}})_{max}$, decrease towards the equilibrium state at the limit $\gamma _{e}\rightarrow 1$, and also they decrease with the temperature of the plasma ions. Then, the allowed domains of the IASWs are shrunk (reduced) with the temperature of the plasma ions.
- Generally, the maximum intervals of the allowed adiabatic Mach numbers and qualified potentials for the propagation of IASWs happen at the asymptotic limits $\gamma _{e} \rightarrow 1$ (the isothermal electrons) and $\sigma _{ie}\rightarrow 0$ (the cold-ion limit), where they are given by $1<{\mathcal {M}}_{\gamma _{e}}<1.58$ and $0<\phi ^{'}_{max}<1.26$.

### Supplementary Information

Supplementary Information.

## Data Availability

Some methods used during this study are included in the “Supplementary Material” file. This article is a theoretical (analytical) study without generating new data. All data used during numerical analysis of this study are addressed in this published article.
