# Supplementary material for: Propagation and domains of the invariant ion-acoustic solitons in the plasmas
Source: Sci Rep. 2024 Feb 13;14:3586. doi: 10.1038/s41598-024-54263-x (PMC10864396; doi:10.1038/s41598-024-54263-x)
Supplement: Supplementary file 1 — Supplementary Information. [file 41598_2024_54263_MOESM1_ESM.pdf]

Supplementary Material for "Propagation and domains of the invariant ion-acoustic solitons in the plasmas"

E. Saberian*
Department of Physics, Faculty of Basic Sciences,
University of Neyshabur, 9319774446, Neyshabur, Iran
e.saberian@neyshabur.ac.ir

THE ESCORT CANONICAL PROBABILITY DISTRIBUTION AND RELEVANT NUMBER DENSITY OF THE KAPPA DISTRIBUTED PARTICLES

In the presence of potential energy, the (escort) canonical probability distribution is written in terms of κ_0 and the total degrees of freedom $d$as follows [1]

$$P(\vec{r},\vec{u};\kappa_0,T) \propto \left[1+\frac{1}{\kappa_0}\cdot\frac{\varepsilon_K(\vec{u})+\Phi(\vec{r})}{k_B T}\right]^{-\kappa_0-1-\frac{1}{2}d}, \tag{1}$$

where, $\varepsilon_K(\vec{u})$ and $\Phi(\vec{r})$ are respectively the kinetic and potential energy of the system, k_B is the Boltzmann constant, and T is the temperature of the system. Here, the Hamiltonian function of the system is $H(\vec{r},\vec{u})=\varepsilon_K(\vec{u})+\Phi(\vec{r})$ and the total degrees of freedom d is defined by the ensemble averaging the Hamiltonian as $\frac{1}{2}d=\frac{\langle H\rangle}{k_BT}$. So, d is the summation of kinetic and potential degrees of freedom as $d=d_K+d_\Phi$, where they are given by $\frac{1}{2}d_K=\frac{\langle\varepsilon_K\rangle}{k_BT}$ and $\frac{1}{2}d_\Phi=\frac{\langle\Phi\rangle}{k_BT}$, respectively. Note that depending on the interaction between the system and the external potential, d_Φ is either positive or negative. We may alternatively define d_Φ to be a positive quantity by using the sign function via the relation $d_\Phi\cdot sign(\Phi)=\frac{\langle|\Phi|\rangle}{k_BT}$, or equivalently by using a suitable cutoff operator.

The connection between κ and q indices in the modern version of canonical probability distribution holds under the transformation $\kappa\equiv\frac{1}{q-1}$ or $q\equiv 1+\frac{1}{\kappa}$ [1]. So, we may rewrite the canonical probability distribution (1) in terms of the zero dimensional index q_0 by exchanging the variables as $\kappa_0=\frac{1}{q_0-1}$. We may also write other versions of the canonical probability distribution in terms of κ_1, κ_2 and κ_3 or equivalently in terms of q_1, q_2 and q_3. Note that depending on the value of d, the canonical probability distribution (1) is a multi-dimensional distribution for handling the κ distribution function.

By calculating the statistical moments of the canonical probability distribution (1) over the velocity space, and after some calculations including the suitable ensemble averaging, one may find the number density of the kappa distributed particles as follows [2]

$$n(\vec{r})=n_\infty\cdot\left[1+\frac{1-\gamma}{\gamma}\cdot\frac{\Phi(\vec{r})}{k_BT_\infty}\right]^{\frac{1}{\gamma-1}}, \tag{2}$$

where n_∞ and T_∞ are the number density and temperature at the infinity. Here, γ is the polytropic index associated with the kappa distributed particles, which is written in terms of κ_0 and d_Φ as follows [2]

$$\gamma=\frac{\kappa_0+\frac{1}{2}d_\Phi}{\kappa_0+\frac{1}{2}d_\Phi+1}. \tag{3}$$

Note that by this formalism, we have the usual thermodynamic evolution of the system as $p(\vec{r})\propto n(\vec{r})^\gamma$, where $p(\vec{r})$ is the thermal pressure of the kappa distributed particles [2].

THE SAGDEEV'S PSEUDO-POTENTIAL FUNCTION IN THE CASE OF WARM PLASMA

Deriving the energy-integral for the warm plasma leads to an integral as $I=\int n'_-(\phi')d\phi'$, where n'_- is the negative branch of Eq.(12) in the main text. We use the change of variable as follows

$$\chi=\frac{\mathcal{M}_{\gamma_e}^2+\frac{3\sigma_{ie}}{Z_i\gamma_e}-\frac{2\phi'}{\gamma_e}}{\sqrt{\frac{12\sigma_{ie}}{Z_i\gamma_e}}\mathcal{M}_{\gamma_e}}, \tag{4a}$$

$$\Theta=\exp(\cosh^{-1}\chi), \tag{4b}$$

where $\chi>0$. Then n'_- is rewritten in terms of Θ as follows

$$n'_-=\frac{\sqrt{\mathcal{M}_{\gamma_e}}}{\left(\frac{12\sigma_{ie}}{Z_i\gamma_e}\right)^{\frac{1}{4}}}\Theta^{-\frac{1}{2}}. \tag{5}$$

Then, the integral I is transformed in terms of Θ, where its solution is

$$I = -\gamma_e \left(\frac{3\sigma_{ie}}{Z_i \gamma_e} \mathcal{M}_{\gamma_e}^6 \right)^{\frac{1}{4}} \left[(\Theta^{\frac{1}{2}} - \Theta_0^{\frac{1}{2}}) + \frac{1}{3} (\Theta^{-\frac{3}{2}} - \Theta_0^{-\frac{3}{2}}) \right]. \tag{6}$$

Inserting I into the Poisson equation in the process of integrating, considering the boundary conditions for having a localized solitary wave, as $\phi^{'}, \frac{d\phi^{'}}{d\xi^{'}}, \frac{d^2\phi^{'}}{d\xi^{'2}} \to 0$ when $|\xi^{'}| \to \infty$, we may find the energy-integral equation for trapping the IASWs in a warm plasma as follows

$$\frac{1}{2} \left(\frac{d\phi^{'}}{d\xi^{'}} \right)^2 + \psi(\phi^{'}, \mathcal{M}_{\gamma_e}; \gamma_e, Z_i, \sigma_{ie}) = 0, \tag{7}$$

where $\psi(\phi^{'}, \mathcal{M}_{\gamma_e}; \gamma_e, Z_i, \sigma_{ie})$ is the Sagdeev's pseudo-potential function as follows

$$\psi(\phi^{'}, \mathcal{M}_{\gamma_e}; \gamma_e, Z_i, \sigma_{ie}) = \gamma_e \left[1 - \left(1 - \frac{1-\gamma_e}{\gamma_e} \phi^{'} \right)^{\frac{\gamma_e}{\gamma_e - 1}} \right]$$
$$-\gamma_e^2 \left(\frac{3\sigma_{ie}}{Z_i \gamma_e} \mathcal{M}_{\gamma_e}^6 \right)^{\frac{1}{4}} \left[(\Theta^{\frac{1}{2}} - \Theta_0^{\frac{1}{2}}) + \frac{1}{3} (\Theta^{-\frac{3}{2}} - \Theta_0^{-\frac{3}{2}}) \right]. \tag{8}$$

Furthermore, change of variable yields the following results

$$\Theta = \chi + \sqrt{\chi^2 - 1}, \tag{9a}$$
$$\Theta^{\pm\frac{1}{2}} = \sqrt{\chi \pm \sqrt{\chi^2 - 1}}, \tag{9b}$$

where it gives Θ in terms of $\phi^{'}$ as follows

$$\Theta = \frac{\mathcal{M}_{\gamma_e}^2 + \frac{3\sigma_{ie}}{Z_i\gamma_e} - \frac{2\phi^{'}}{\gamma_e}}{\sqrt{\frac{12\sigma_{ie}}{Z_i\gamma_e}} \mathcal{M}_{\gamma_e}} + \sqrt{\frac{\left(\mathcal{M}_{\gamma_e}^2 + \frac{3\sigma_{ie}}{Z_i\gamma_e} - \frac{2\phi^{'}}{\gamma_e} \right)^2}{\frac{12\sigma_{ie}}{Z_i\gamma_e} \mathcal{M}_{\gamma_e}^2} - 1}, \tag{10}$$

[1] G. Livadiotis and D. J. McComes, The Astrophysical Journal 88, 741 (2011).
[2] G. Livadiotis, The Astrophysical Journal 874, 10 (2019).
